# Inadvertent Wire Shearing During Endoscopic Ultrasound-Guided Rendezvous Procedure

**DOI:** 10.14309/crj.0000000000001573

**Published:** 2024-12-20

**Authors:** Karam Karam, Elias Fiani, Ihab I. El Hajj

**Affiliations:** 1 Department of Gastroenterology and Hepatology, University of Balamand, Beirut, Lebanon; 2 Department of Gastroenterology and Hepatology, Saint George University of Beirut, Beirut, Lebanon

**Keywords:** EUS, rendezvous procedure, pancreatography, adverse event, wire shearing

## CASE REPORT

**Figure 1.**
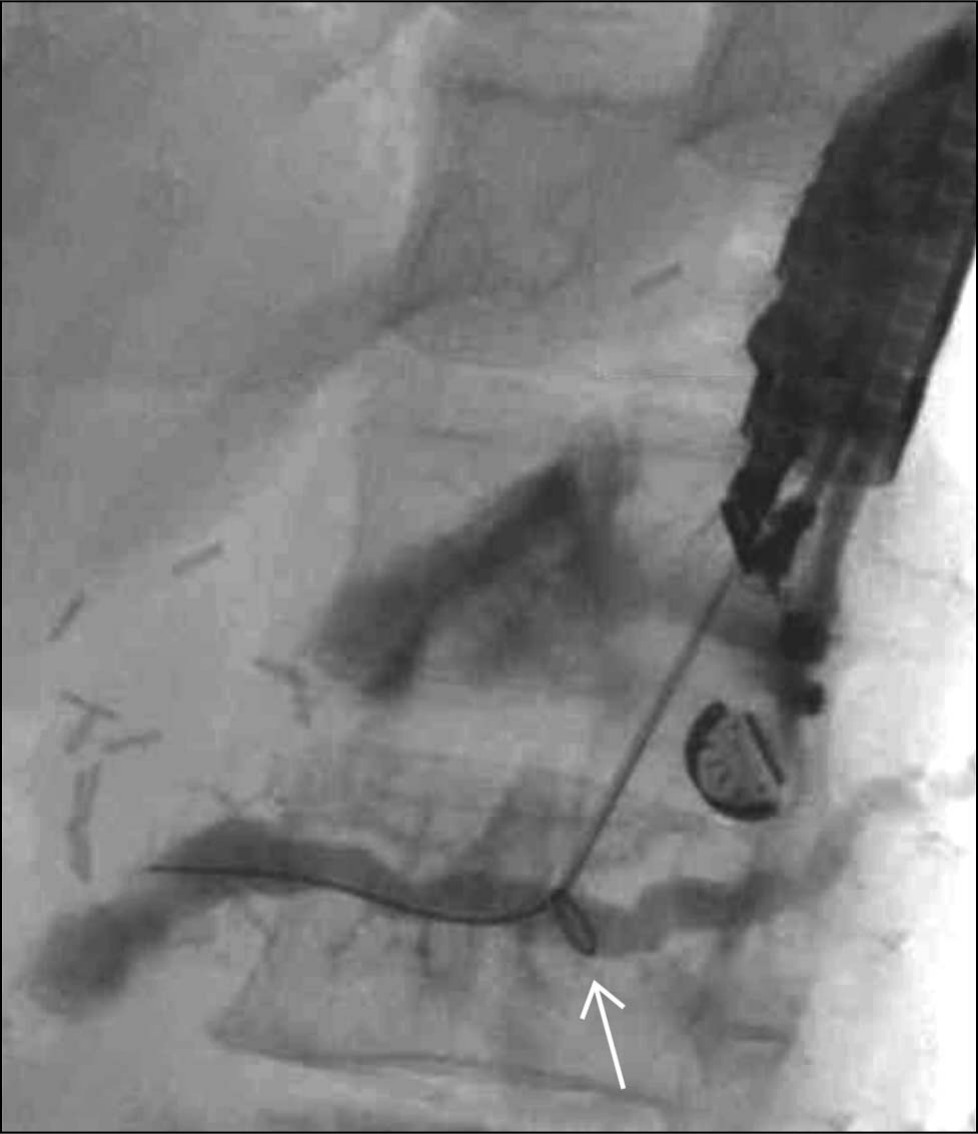
Wire manipulation after endoscopic ultrasound-guided puncture of the main pancreas duct resulted in formation of an alpha loop within the main pancreas duct (arrow).

**Figure 2.**
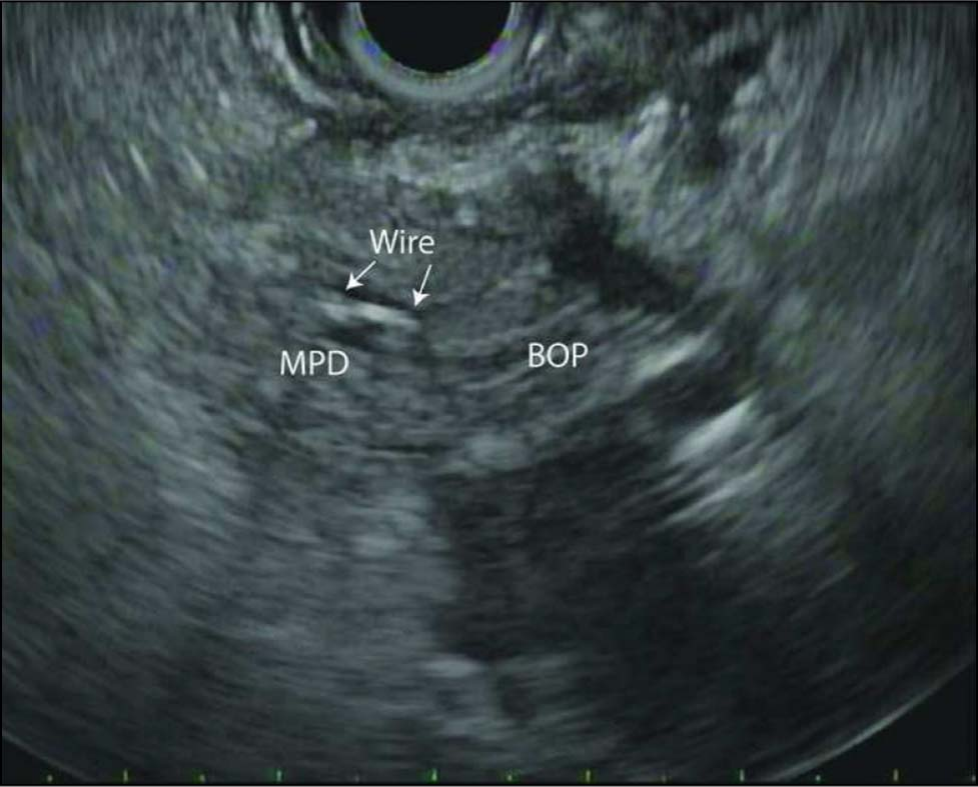
Endoscopic ultrasound showing the retained wire sheath (arrows) within the lumen of the main pancreas duct. MPD, main pancreas duct.

**Figure 3.**
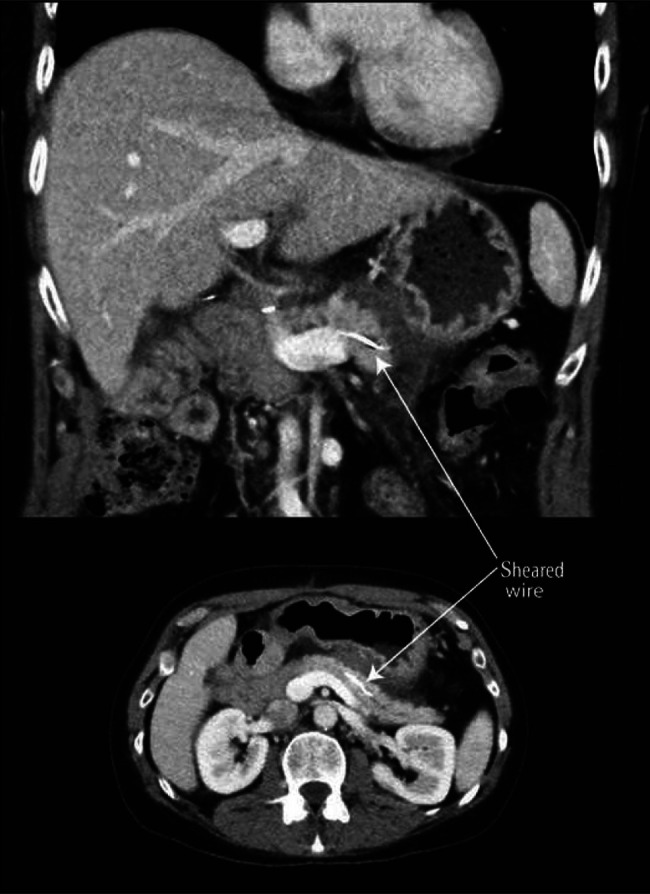
Computed tomography scan (coronal and axial views) identified the sheared wire within the lumen of the main pancreas duct.

A 46-year-old woman presented with recurrent acute pancreatitis 3 years after pylorus preserving pancreaticoduodenectomy for chronic pancreatitis. Magnetic resonance imaging/magnetic resonance cholangiopancreatography showed pancreaticojejunal (PJ) anastomosis stricture and dilated main pancreas duct (MPD) at 6 mm. Endoscopic ultrasound (EUS)-guided rendezvous was scheduled. A 19 G ExpectTM needle (Boston Scientific, Marlborough, MA) was used to puncture the MPD in the body of the pancreas. EUS-guided pancreatography confirmed chronic pancreatitis. A straight 0.025 inch VisiGlide wire (Olympus America, Westborough, MA) was advanced through the fine needle aspiration (FNA) needle into MPD antegrade toward the PJ anastomosis. Multiple attempts at traversing the PJ anastomosis were unsuccessful. Wire manipulation resulted in formation of an alpha loop within the MPD (Figure 1). Withdrawal of the wire resulted in formation of a coil of sheared wire beyond the needle tip, identified on fluoroscopy and live EUS (Figure 2). A second duct puncture was made. A 0.018 inch NovaGold wire (BSC) was advanced with the intent of traversing and then pushing the retained wire across the PJ stricture. Multiple attempts were unsuccessful. CT scan showed the retained wire sheath (Figure 3), and no other complications. The patient underwent surgical revision of the PJ anastomosis and removal of the sheared wire.

Shearing of the sheath of the Guidewire used during EUS-guided therapy (rendezvous procedure, biliary or pancreatic drainage), is a rare—yet serious—complication and limited to case reports.^1–3^ Endoscopic management depends on the expertise of the endoscopy team and the availability of special endoscopy tools (blunt-ended access needle [Cook Medical, Winston Salem, NC]).^1,2^ Alternative treatment involves the interventional radiology team or the surgery team. Endoscopists should be aware of this complication and seek to avoid it by minimizing endoscope torque, repeated elevator deflection, excessive needle bending, and wire manipulation, and by carefully inspecting for wire structural integrity during the procedure.

## DISCLOSURES

Author contributions: K. Karam prepared the manuscript and reviewed the literature. E. Fiani reviewed the manuscript. II El Hajj reviewed the manuscript, and is the article guarantor.

Financial disclosure: None to report.

Informed consent was obtained for this case report.
